# FP10 – the Competitiveness Fund for Europe

**DOI:** 10.1038/s44319-025-00462-0

**Published:** 2025-04-24

**Authors:** Liselotte Højgaard

**Affiliations:** University of Copenhagen, Rigshospitalet, and DTU The Technical University of Denmark, Copenhagen, Denmark

**Keywords:** Economics, Law & Politics, Science Policy & Publishing

## Abstract

The planned Competitiveness Fund for Europe should not come at the expense or jeopardise the quality standards of the highly successful EU scientific Framework Programme.

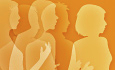

This year, I had promised myself not to get into trouble. However, I then decided to accept the invitation from *EMBO Reports* to comment on the European Commission’s plans for a Competitiveness Fund for Europe. In essence, Commission President Ursula von der Leyen proposes to combine different funding instruments— including EU research programs—into a “European Competitiveness Fund”. These plans, though not detailed, have prompted the European Parliament and EU science ministers to issue the ‘Warsaw Declaration’, stating that the Framework Program for research should remain a stand-alone scheme. Apart from noting a lack of transparency and consultation with parliament and other stakeholders, critics fear that the decision-making processes of the Framework Programme and the ERC that are purely based on merit and excellence could be replaced with political criteria, which would jeopardize the enormous success of the EU’s science-funding schemes.

In commenting on this, I have reflected on my experiences from 30 years in science policy. Alongside being a clinician scientist, I have been editor of the Danish medical journal, and a member of The International Committee of Medical Journal Editors, the “Vancouver Group”. I was part of the European Strategy Forum on Research Infrastructure, sketching the first ESFRI Roadmap for the life sciences. As Chair of EMRC, the European Medical Research Council, I was involved in FP 7, Horizon 2020 and Horizon Europe. I have served as Chair of the Danish National Research Foundation, member of INSERM, die Robert Bosch Stiftung, Novo Nordisk Foundatio and from 2020 on the Scientific Council of ERC. Yes, I am a dinosaur.

ERC has been a success since it was established in 2007. The mission is to encourage the highest quality in research through competitive funding based on excellence, by supporting investigator-driven frontier research across all fields. The annual budget from 2021 to 2027 is €16 billion, or 17% of the Horizon Europe budget. ERC has funded 16,000 projects, more than 100,000 researchers, and evaluated more than 130,000 research proposals with the best researchers serving on the evaluation panels. ERC projects have led to 2200 patents and 400 start-ups, 200,000 articles in scientific journals, and ERC grantees have received 14 Nobel Prizes, 7 Fields Medals, and dozens of other important prizes. Among the ERC-funded life-science projects, 64% led to quotations in patent applications. ERC-funded research was behind the Covid-19 vaccines. This is an amazing track record: it demonstrates that a high fraction of frontier research directly leads to innovation.

In Europe, at least, nobody seems to question the type of research funded by the ERC: frontier research is acknowledged as the basis for the solutions of the future. The reports from Mario Draghi, Enrico Letta and Manuel Heitor all emphasise the importance of research, innovation, and education for the future of Europe, including the strengthening of the ERC. Letta’s report “Much more than a market” proposes research, innovation, and education as a fifth amendment to the European Open Market. Draghi’s report “The future of European Competitiveness” stresses the importance of research and innovation to mitigate the challenges ahead of us and emphasizes that Europe should strive for world-class research. CERN, EMBL, and many universities and research organizations in Europe are doing well, but we are not at the very top, and we are still missing out on innovation. If basic research were to be diminished for the sake of a Competitiveness Fund, it would be at odds with the recommendations of the three reports.

Having visited many US research institutions throughout the years, I have been impressed by their size, infrastructure, equipment, collaboration, financial support, and organization—and all the great minds gathered. It is all about giving people driven by curiosity the freedom, flexibility, trust, and support they need. With a dedicated long-term effort to support the best and most brilliant, it can also be done in Europe. The ERC has paved the way, while the European Innovation Council (EIC) is on the right track. Of course, Europe could further benefit by a straight highway leading from ERC to EIC.

To take innovation to the next level, a network of European Innovation districts could strengthen the concept. By collaborating and sharing best practices, we will succeed faster, instead of wasting time, energy, brains and money on futile competition and mishaps. Such innovation networks should imitate the Kendall Square model from Boston, with Harvard, MIT, the Harvard hospitals, and the biotech industry working together. In Denmark, the University of Copenhagen, The Technical University of Denmark, Rigshospitalet and the other university hospitals, and most importantly the life-science industry and quantum-research ecosystem are establishing an “Innovation District Copenhagen” with BioInnovation Institute (BII) at its center. A hub and spoke model connecting such innovation districts in Europe could make a shortcut to success through collaboration and friendly competition.

Niels Bohr had a horseshoe hanging over the door of his summerhouse and was questioned about whether he believed in it. His reply was: “It works also if you don’t believe in it”. I am not superstitious, but worried about abolishing the Framework Program in these difficult times just before a renewal of FP10. It has served us well over the decades and has been developed into a refined instrument. It was therefore encouraging to hear in early April 2025 that EU Commissioner Ekaterina Zaharieva noted that the EU can have both: the European Competitiveness Fund and an independent, stand-alone Framework Program for research and innovation. It might be a great solution, but only if they do not end up cannibalizing each other or from other areas in need of funding—be it energy, defense, security or agriculture. In such a scenario, research would surely suffer. It takes decades to build a flourishing research area, but it might take only a few weeks to undermine it. The current events on the other side of the Atlantic should be a warning for EU science politics.

When I was a member of the Danish Council on Research Policy, the then old dinosaurs from agriculture were less inclined to support medical research. Every time medical research groups were quarreling publicly, they arrogantly pointed at me and said “Ha, there you lost 100 million”. The Danish position paper therefore advocates for balance. Not Technology Readiness Level 9–10 type of research against 1–2 type research; not the humanities against the natural sciences, but all together with a focus on interdisciplinarity, quality, and internationalization. Quarrels over whether to call it “FP-10 and RTD” or “Competitiveness Fund”, or, worse, competition between the two may lead to diminished overall budgets and, in the end, sideline the concept of research and innovation.

Many key stakeholders defend research at this time, which is highly appreciated. If nobody defends research, then budgets will be cut and programs will deteriorate. If turf battles were not fought, we would be lagging behind the USA, China, Japan and others. If the researchers, universities, alliances, academies, foundations, LERU, EMBO, and all the other key stakeholders had not spend time and energy to defend basic research, we would have lost.

A strong and independent ERC is therefore a *sine qua non* as a basis for innovation. It has been refined to perfection throughout the last 16 years, and we are still working on it to make it even better. The FP-10 calls can be simplified and reduced in complexity, and I propose an “Omnibus” to simplify conditions for and organization of research, and another Omnibus to cut red tape and support industry. The Marie Sklodowska Curie stipends to support young scientists have been a huge advantage for Europe as many big discoveries were made by young scientists. The collaborative applied research supported by Pillar II has been a tremendous leverage too by spreading knowledge and sharing best practices.

The huge contemporary challenges—human and planetary health, climate change, sustainability, and securing food production—need our full attention. Frontier research is essential as the basis for applied research and innovation that will provide solutions. Europe is at a crossroads. If we are wise, and we have been periodically, we team up and use research to meet the challenges.

If we call the next solution “FP-10—The European Competitiveness Fund” exploiting and combining the good elements of the many previous framework programs without cannibalizing each other, it would kill two birds with one stone; or actually the opposite, killing no birds but making European research the immortal Phoenix of tomorrow. Reinvigorating European research and innovation should be the most important goal.

## Supplementary information

Peer Review File

