# Supplementary material for: FP10 – the Competitiveness Fund for Europe
Source: EMBO Rep. 2025 Apr 24;26(10):2485–6. doi: 10.1038/s44319-025-00462-0 (PMC12116929; doi:10.1038/s44319-025-00462-0)
Supplement: Supplementary file 1 — Peer Review File [file 44319_2025_462_MOESM1_ESM.pdf]

EMBO reports

FP10 - The Competitiveness fund for Europe

Liselotte Hoejgaard

Corresponding author(s): Liselotte Hoejgaard (liselotte.hoejgaard@regionh.dk)

Review Timeline:

Submission Date:	16th Apr 25
Accepted:	17th Apr 25

Editor: Holger Breithaupt

Transaction Report: This article type is not peer reviewed.
